# Clinical characterization and prognosis of T cell acute lymphoblastic leukemia with high *CRLF2* gene expression in children

**DOI:** 10.1371/journal.pone.0224652

**Published:** 2019-12-12

**Authors:** Mingmin Wang, Jinquan Wen, Yuxia Guo, Yali Shen, Xizhou An, Yanni Hu, Jianwen Xiao

**Affiliations:** 1 Department of Hematology, Children’s Hospital of Chongqing Medical University, Chongqing, P.R. China; 2 Ministry of Education Key Laboratory of Child Development and Disorders, Chongqing, P.R. China; 3 Chongqing Key Laboratory of Pediatrics, Chongqing, P.R. China; 4 Department of Hematology, Children’s Hospital of Xianyang, Xi’an, P.R. China; 5 China International Science and Technology Cooperation Center for Child Development and Disorders, Chongqing, P.R. China; The University of Adelaide, AUSTRALIA

## Abstract

It has been reported that overexpression of the *CRLF2* gene is associated with poor outcomes in pediatric B cell acute lymphoblastic leukemia (B-ALL), but the incidence rates, clinical characteristics and outcomes of *CRLF2* gene overexpression in pediatric T cell ALL (T-ALL) have not been systematically analyzed. In this study, CRLF2 mRNA expression levels and clinical and laboratory parameters in 63 pediatric T-ALL patients were detected at the Children's Hospital of Chongqing Medical University and Children’s Hospital of Xianyang between February 2015 and June 2018. The patients were treated according to the modified St. Jude TXV ALL protocol, and early treatment responses (bone marrow smear and MRD level) and prognoses in the enrolled patients were assessed. *CRLF2* overexpression was detected in 21/63 (33.33%) patients. Statistical differences were not found for clinical or laboratory parameters (including sex, age, initial WBC count, incidence mediastinal involvement, abnormal karyotype and fusion genes) between patients with high *CRLF2* expression and patients with low expression of *CRLF2* (*P*>0.05). One patient died of tumor lysis syndrome and renal failure, and the treatment response was monitored on day 19 (TP1) of remission in 62 patients. One patient quit treatment because of family decisions, and 61 patients underwent treatment response evaluation on day 46 (TP2) of remission. Significant differences were not found between patients with high *CRLF2* expression and patients with low *CRLF2* expression in terms of the treatment responses at TP1 or TP2 (*P*>0.05). Following October 2018, 12 patients among the 61 evaluable patients relapsed (relapse rate: 19.67%), 3 patients died from chemotherapy, and the treatment-related mortality (TRM) rate was 4.92%. Secondary tumors occurred in 1 patient. The 3-year prospective EFS rate was 54.1±11.2% and 77.7±6.6% for the 61 evaluable patients and 58 patients without TRM. Patients with low *CRLF2* expression had longer EFS durations than patients with high *CRLF2* expression (61 evaluable patients: 35.91± 2.38 months vs 23.43± 2.57 months; 58 patients without TRM: 37.86± 2.08 months vs 24.55±2.43 months, *P*<0.05). *CRLF2* expression levels were also monitored in 13 patients at TP1 and TP2, and the MRD level did not vary with the *CRLF2* expression level. Our data suggest that clinical features, laboratory findings and treatment responses in the pediatric T-ALL population do not vary based on the overexpression of *CRLF2* but that *CRLF2* overexpression can contribute to a high risk of relapse in pediatric T-ALL. Thus, *CRLF2* expression levels should not be used as biomarkers for monitoring MRD.

## Introduction

T cell acute lymphoblastic leukemia (T-ALL) accounts for approximately 15–20% of ALL in children [1]. Despite improvements in intensive chemotherapy and allogenic hematopoietic stem cell transplantation (allo-HSCT), 15–20% and 20–40% of pediatric T-ALL patients die of relapse or treatment failure in developed and developing countries, respectively [2,3]. The Cytokine receptor-like factor 2 (*CRLF2*) gene encodes a member of the type I cytokine receptor family, and the encoded protein is a receptor for thymic stromal lymphopoietin (*TSLP*) [4]. Together with the interleukin 7 receptor (IL7R), the CRLF2 protein and *TSLP* activate the *STAT3*, *STAT5*, and *JAK2* pathways, which control processes such as proliferation of cells and development of the hematopoietic system [5,6]. Rearrangement of this gene with immunoglobulin heavy chain gene (IGH) on chromosome 14 or with P2Y purinoceptor 8 gene (*P2RY8*) on the same X or Y chromosome is associated with B-progenitor ALL (B-ALL) [7] and Down syndrome-related ALL [8].

Overexpression of *CRLF2* in B-ALL has been reported and shown to be correlated with poor outcomes in pediatric and adult B-ALL [7,9]. However, the correlation between *CRLF2* expression level and the clinical features and prognoses of T-ALL patients has not been thoroughly investigated [10]. This study prospectively assessed the influence of *CRLF2* overexpression on the clinical features and prognoses of T-ALL in children and adolescents.

## Materials and methods

### Patients

A total of 63 newly diagnosed T-ALL (aged ≤15 years) patients were enrolled in this study. The patients were treated following a modified St. Jude TXV protocol [11] (S1 Table) at the Children's Hospital of Chongqing Medical University (CHCMU) and Children’s Hospital of Xianyang between February 2015 and June 2018. Patients who were diagnosed with *BCR/ABL1*-positive T-ALL, early T precursor ALL (ETP-ALL), or secondary leukemia or patients who had received chemotherapy before hospitalization were excluded. Ethical approval for the treatment was granted by the Ethics Commission of CHCMU, and informed consent was obtained from the patients or their guardians. Clinical features, laboratory findings and prognostic data for the enrolled patients were collected and analyzed from clinical records.

The initial diagnosis of T-ALL was based on FAB morphological classification detected by cytomorphological observation in BM smears and immuno-phenotype detection by flow cytometry (FCM) according to protocol [11]; chromosomal karyotyping and fluorescence in situ hybridization (FISH) of chromosomal translocation including *ETV6/RUNX1*, *MLL* rearrangements (*MLLr*), *BCR/ABL* and *TCF3/PBX1* were analyzed as reported in the literature [12]. Twenty common fusion genes, including *ETV6/RUNX1*, *TCF3/PBX1*, *TCF3/HLF*, *BCR/ABL1*, *MLL/AF10*, *MLL/AF4*, *MLL/AF9*, *MLL/ENL*, *MLL/AF1p*, *MLL/AF1q*, *MLL/AF6*, *MLL/AFX*, *dupMLL*, *SIL/TAL1*, *ETV6/ABL1*, *SET/NUP214*, *EBF1/PDGFRB*, *TLS/ERG*, *SET/CAN* and *HOX11*, were detected by multiplex nested reverse transcription polymerase chain reaction (multiplex RT-PCR), and positive results were confirmed by split RT-PCR as reported in the literature [13,14].

### Treatment protocol and therapeutic evaluation

The protocol was divided into four phases: remission induction, consolidation, continuation and maintenance (S1 Table); patients received triple intrathecal injection (TiT) and high-dose methotrexate (HD-MTX) for prophylaxis of central nervous system (CNS) involvement following the protocol.

Early treatment responses based on cytomorphological detection of bone marrow (BM) were conventionally conducted at two time points (TP): TP1 (day 19 of remission induction) and TP2 (day 46 of remission induction). BM smear status was recorded as in [11]: M1 (<5% leukemic cells) or complete remission (CR), M2 (5–25% leukemic cells), and M3 (≥25% leukemic cells). Minimal residual disease (MRD) level was detected by FCM and described according to the literature report [11]. MRD level of ≥1×10^−4^ was considered positive.

Central nervous system leukemia (CNSL) or testicular leukemia (TL) were defined and recorded as in the literature [15]. Event was defined as each of the following situations [11]: refractory disease (BM status M2 or M3 at TP2), relapse (BM relapse and/or extramedullary relapse), death (any reason) or diagnosis of secondary malignancy.

### Detection of *CRLF2* expression levels

Total RNA from BM samples was converted to cDNA, and the expression of *CRLF2* was detected by real-time quantitative polymerase chain reaction (qRT-PCR) assays and performed as reported in the literature [10]. Expression of the gene Eukaryotic translation elongation factor 2 (*EEF2*) was chosen as an internal control. The ^δ^Ct value was calculated and the expression ratio was defined by the percentage of ^δ^Ct value in the ^δ^Ct table of *CRLF2* of a standard cohort from the detection laboratory. High expression of *CRLF2* was defined as an expression ratio ≥90% and low expression of *CRLF2* was defined as an expression ratio<90% [9].

### Statistical analysis

Following October 2018, data on the clinical features, laboratory findings, treatment responses, treatment-related mortality (TRM) and event-free survival (EFS) rates of these patients were collected and analyzed. EFS was calculated from the date of diagnosis to the last follow-up, loss of follow-up or loss of the first event. SPSS 19.0 (IBM Corp., Armonk, NY) software was employed for statistical analysis. Survival curves were plotted according to the Kaplan-Meier method and compared using the log-rank test. Proportional differences between patient groups were analyzed by Pearson’s chi-square (χ^2^) tests or Fisher's exact tests. A *P* value <0.05 was regarded as significantly different.

## Results

### Clinical features of the whole cohort

Data on clinical characteristics, including age at diagnosis, sex, white blood cell (WBC) count in peripheral blood, chromosomal karyotype, existence of fusion genes and mediastinal involvement, were collected and are described in Table 1. Of the 63 patients enrolled in this study, 50 were male and 13 were female. The age at diagnosis of the enrolled patients ranged from 19 months to 178 months (median age: 97.22± 5.71 months; average age: 97.22± 5.71 months), and 23 (36.51%) patients were more than 10 years old. The initial WBC count was 1.05–543.79×10^9^/L (median value: 62.51×10^9^/L; average value: 131.41± 19.42×10^9^/L); 24 (38.10%) patients had initial WBC counts ≥ 100×10^9^/L, and 15 (23.81%) patients had initial WBC counts <10×10^9^/L. All 63 patients underwent karyotype detection, and normal karyotype was found in 48 patients, whereas abnormal karyotype was found in 12 patients. Samples from 3 patients could not be analyzed due to the lack of mitotic cells. The DNA index was all ≥1.0 for the 60 analyzed patients. Recurrent cytogenetics and molecular genetic features were determined by FISH and multiplex RT-PCR, respectively: 44 (69.84%) patients were negative; *SIL/TAL* fusion gene was positive in 16 (25.40%) patients; *BCR/ABL1*, *SET/CAN* and *MLL/ENL* fusion genes were detected in 1 patient. TL and CNSL were also confirmed in 1 patient. Chest CT scans were performed, and mediastinal involvement was found in 37 (58.73%) cases.

**Table 1:** Comparison between cases with high and low *CRLF2* expression based on clinical features.

Feature	High expression of *CRLF2*	Low expression of *CRLF2*	*t* or Chi-square value	*P* value
Age (months)	103.43±11.72	94.12±6.30	*t* = 0.7661 df = 61	0.4465
Sex (male:female)	14:6	36:7	χ^2^ = 1.5691	0.2103
Mediastinal mass (n, %)	11 (55.00%)	28 (65.12%)	χ^2^ = 0.59241	0.4415
WBC count (×10^9^/L)	175.5 ± 41.94	109.4 ± 19.77	*t* = 1.626 df = 61	0.1091
Abnormal karyotype (n, %)	4 (20.00%)	10 (25.00%)*	χ^2^ = 0.16801	0.6819
Positive fusion gene (n, %)	6 (30.00%)	12 (27.91%)	χ^2^ = 3.0641	0.0800
SIL/TAL fusion gene (n, %)	6 (30.00%)	10 (23.26%)	χ^2^ = 0.32771	0.5670

*: Three patients failed karyotype analysis.

In this study, 42 (66.67%) patients presented with low *CRLF2* expression, and 21 (33.33%) patients presented with high *CRLF2* expression. Clinical characteristics were compared between patients grouped by high and low *CRLF2* expression (Table 1). Statistical differences were not found based on sex, age at diagnosis, initial WBC count, mediastinal involvement, karyotype, chromosomal translocations or *SIL/TAL* fusion gene between the two groups.

### Treatment responses and prognosis

The 63 enrolled patients received treatment according to a modified St. Jude TXV protocol. Except for 1 patient who died of tumor lysis syndrome and secondary renal failure during the 1^st^ week of remission induction, BM smears and MRD levels were recorded for 62 patients at TP1. Cytomorphological detections of BM smears showed 53 (85.48%), 6 (9.68%) and 3 (4.84%) patients with BM status M1, M2 and M3, respectively. MRD level was also detected by FCM at TP1; 29 (46.77%) patients had MRD level <1×10^−4^ (negative MRD), and 33 (53.23%) patients had MRD level ≥1×10^−4^ (positive MRD). Among the 33 patients who presented with positive MRD, 7 patients had MRD levels <1%, 10 patients had MRD levels 1%-10%, and 16 patients had MRD levels ≥10%. One patient quit treatment because of family decisions, and BM smears and MRD levels were evaluated in 61 patients at TP2. All 61 patients achieved CR; MRD level was also detected; 51 (83.61%) patients were MRD-negative, 10 (16.39%) patients were MRD-positive, and 4 of the 10 patients with positive MRD had MRD levels <1%, and 6 had MRD levels 1%-10% (Table 2).

**Table 2:** Comparison between cases with high and low *CRLF2* expression based on treatment responses or outcomes.

Feature	High expression of *CRLF2*	Low expression of *CRLF2*	*t* or Chi-square value	*P* value
BM smear (M1:M2+M3) at TP1	15:5	38:3	χ^2^ = 3.689	0.0548
MRD level at TP1 (positive rate, %)	11:9	20:21	χ^2^ = 0.2081	0.6483
MRD level at TP2 (positive rate, %)	5:15	5:36	χ^2^ = 1.608	0.2048
EFS (months)	23.43±2.57	35.91±2.38	χ^2^ = 4.646	0.0311
EFS without TRM (months)	24.55±2.43	37.86±2.08	χ^2^ = 5.496	0.0191

**Fig 1:**
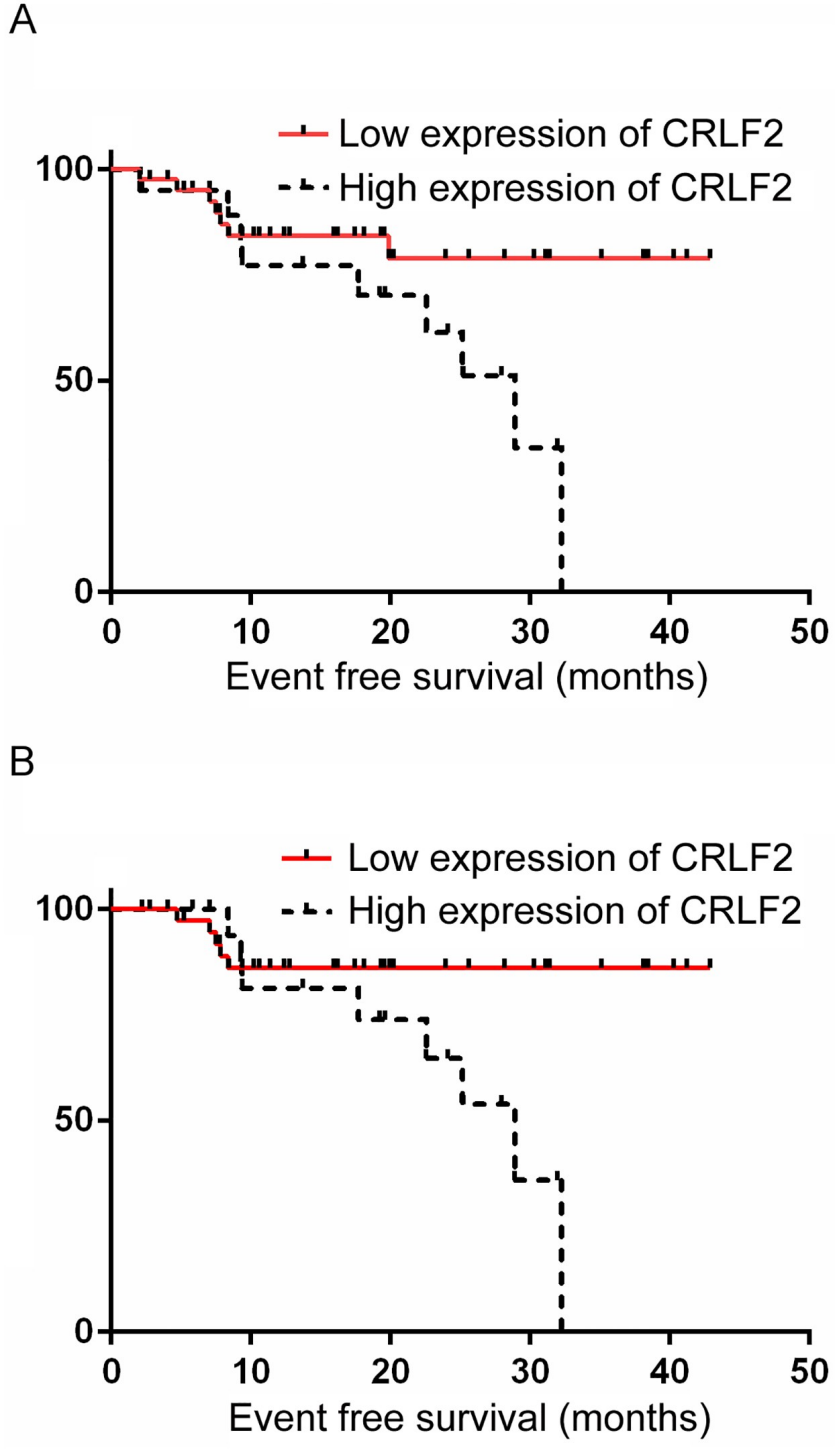
The event free survival curves of the whole cohort.

Following October 2018, among the 61 evaluable patients, 3 patients died of sepsis due to myelosuppression caused by chemotherapy, and the TRM rate was 4.92%; 12 patients relapsed (CNS relapse: 5 patients; BM relapse: 4 patients; combined CNS and BM relapse: 2 patients; and lymph node relapse: 1 patient), and the relapse rate was 19.67%. Langerhans cell histiocytosis occurred in 1 patient, and 3 patients were lost to follow-up after treatment. The three-year prospective EFS (EFS) rate for the 61 evaluable patients was 54.1±11.2%, and the mean EFS duration for the 61 patients was 31.80± 2.25 months (95% CI: 27.40–36.20 months, Fig 1); The three-year EFS rate for the 58 patients who did not die from chemotherapy (without TRM) was 77.7±6.6%, and the mean EFS duration for the 58 patients without TRM was 33.34± 2.20 months (95% CI: 29.02–37.66 months, Fig 1).

**Fig 2:**
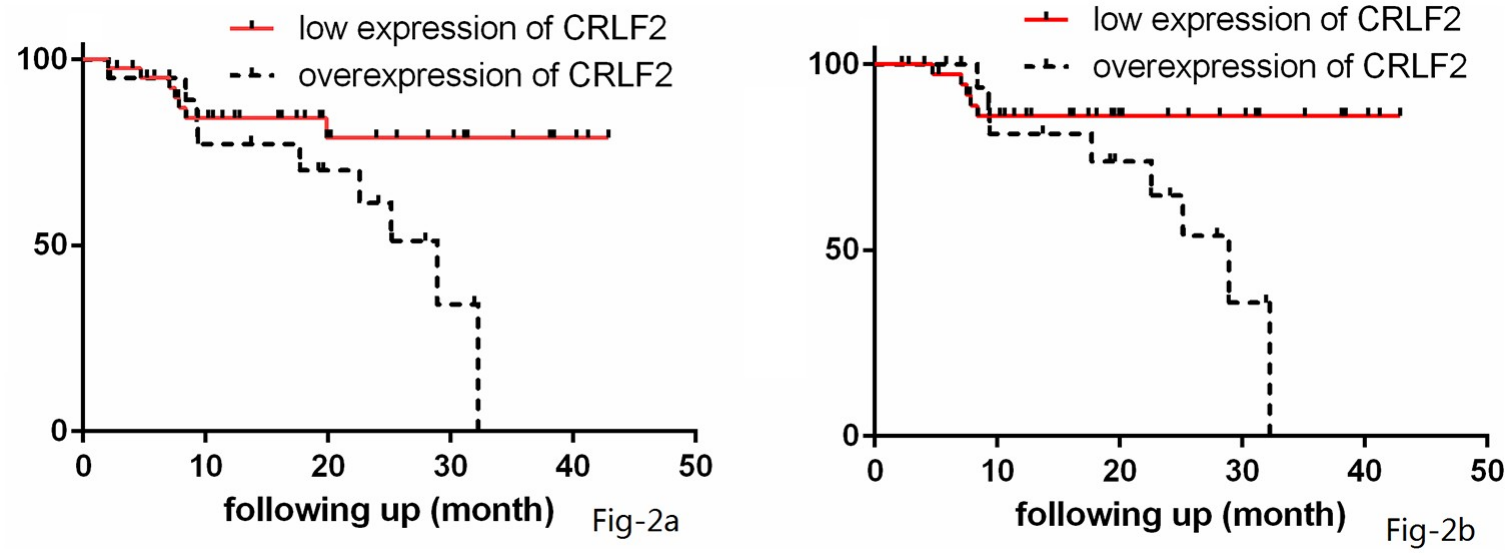
The event free survival curves of cohorts grouped by *CRLF2* expression.

The 63 patients were classified by *CRLF2* expression levels including 42 patients with low *CRLF2* expression and 21 patients with high *CRLF2* expression. The *CRLF2* overexpression rate was 33.33%. Early response indices for chemotherapy (BM smears or MRD levels) were evaluated at TP1 and TP2, and significant differences were not found between patients with high *CRLF2* expression and patients with low *CRLF2* expression. The outcomes of the patients were also analyzed and are demonstrated in Table 2 and Fig 1. Patients with low *CRLF2* expression had longer EFS durations (35.91± 2.38 months vs 23.43± 2.57 months) than patients with high *CRLF2* expression (*P*<0.05, Table 2 and Fig 2a). One patient and 2 patients died from treatment in the low *CRLF2* expression and high *CRLF2* expression group, respectively. For the 58 patients without TRM, the EFS duration in the low *CRLF2* expression group (39 patients) was also superior to that in the high *CRLF2* expression group (19 patients) (37.86± 2.08 months vs 24.55± 2.43 months, *P*<0.05, Table 2 and Fig 2b).

At TP1 and TP2, *CRLF2* expression levels were monitored in 13 patients, including 4 patients with low *CRLF2* expression 9 patients with high *CRLF2* expression at diagnosis. The MRD level did not vary with the *CRLF2* expression level (Table 3). *CRLF2* expression level was also detected in BM samples from 5 of the 13 relapsed patients, and no clear relationships were observed between *CRLF2* expression levels and prognoses at TP1 or TP2 or the relapse status (Table 3).

**Table 3:** *CRLF2* expression level after chemotherapy.

Pt.	At diagnosis		TP1			TP2		
	*CRLF2* level	*CRLF2* expression (%)	MRD	*CRLF2* level	*CRLF2* expression (%)	MRD	*CRLF2* level	*CRLF2* expression
6	low	67.55	positive	high	94.32	positive	low	89.76
8	high	92.78	positive	high	97.31	positive	high	91.09
15	high	91.28	positive	low	44.33	negative	low	80.58
16	high	92.17	positive	low	79.20	negative	low	66.26
17	high	92.77	positive	low	82.99	negative	low	42.29
20	high	95.33	positive	low	44.32	negative	low	62.11
23	low	22.57	positive	low	80.68	negative	high	94.32
26	low	69.28	positive	low	86.10	negative	low	50.94
32	high	92.17	negative	low	65.86	negative	low	67.56
33	high	92.58	negative	low	62.50	negative	low	76.85
34	high	95.18	negative	low	40.69	negative	low	87.42
37	high	91.57	negative	low	57.64	negative	low	73.16
42	low	71.69	negative	low	72.82	negative	low	64.67

Pt.: patient; a MRD value≥0.0001 was positive, MRD value<0.0001 was negative.

## Discussion

T-ALL is a neoplasm of immature T-cell lymphoblasts and is characterized by several genetic alterations and poor prognoses [16]. T-ALL accounts for approximately 15–20% and 20–25% of newly diagnosed ALL cases in children and adults, respectively [1, 17]. T-ALL is an aggressive hematologic malignancy, and outcomes for T-ALL have historically remained poor [1]. With intensive chemotherapy and allo-HSCT, the EFS rates for T-ALL have increased to 85% in developed countries[16,17], whereas the outcomes of T-ALL in developing counties have remained poor. The 3-year EFS rate for this study was 77.7±6.6%, which is similar to that in the literature [3]. The lower EFS rate in our study than that in developed countries may be partially due to the enrolled patients receiving a lower intensity of chemotherapy than the intensive protocols used for T-ALL patients in developed countries [16].

Somatic mutations in the *CRLF2* gene mediated via juxtaposition to the immunoglobulin heavy chain gene (IGH) transcriptional enhancers are present in 7% of B-ALL cases [7] and 50% of DS-ALL patients [8]. *TSLP*, the encoded protein, activates the *STAT3*, *STAT5*, and *JAK2* pathways, which participate in processes of lymphocyte proliferation and development [4–6]. It has been demonstrated that the overexpression of *CRLF2* results in poor outcomes in B-ALL [9]. The incidence rates, clinical features and prognoses of the T-ALL in patients with *CRLF2* overexpression have not been fully discussed [10].

In this study, 33.33% of pediatric T-ALL patients presented with *CRLF2* overexpression, which was higher than that in B-ALL (7%) [7]. Clinical characteristics in patients with high *CRLF2* expression, including sex, age and WBC count at diagnosis, incidence of mediastinal involvement, chromosomal abnormalities and fusion genes were similar between patients with low and high *CRLF2* expression, suggesting that differences in traditional risk factors did not vary according to *CRLF2* expression in T-ALL patients.

It has been well reported that a good therapeutic response is related to favorable outcomes in T-ALL, and a poor BM smear status (M2 or M3) or high MRD level (≥1%) at TP1 or TP2 indicates poor prognosis [11]. The pediatric T-ALL cohort in this study was classified by *CRLF2* expression levels, and the therapeutic response was evaluated. Significant differences in BM smears and MRD levels at TP1 and TP2 were not observed between patients with high and low *CRLF2* expression, suggesting that *CRLF2* overexpression was unrelated to the treatment response in pediatric T-ALL patients. The survival data for this cohort (all evaluable patients and patients without TRM) were also analyzed, and patients with low *CRLF2* expression presented with longer EFS durations than patients with high *CRLF2* expression. These data suggest that *CRLF2* overexpression plays an important role in the outcomes of T-ALL.

*CRLF2* expression levels were monitored at TP1, TP2 or relapse in 13 patients who received chemotherapy, and no relationship was found among MRD levels, prognoses and *CRLF2* expression levels at TP1, TP2 or relapse. This result revealed that more samples and further studies are needed before concluding *CRLF2* expression level as a risk factor for therapeutic response.

In general, in this study, we evaluated the clinical features, treatment responses and prognoses of pediatric T-ALL patients. Our data suggest that clinical features, laboratory findings and treatment responses in the pediatric T-ALL population do not differ according to the expression level of *CRLF2* but that *CRLF2* high expression at diagnosis can contribute to a high risk of relapse in pediatric T-ALL patients. *CRLF2* expression detection at diagnosis might be a good supplement to evaluate the risk stratifications of T-ALL but this index should not be used as biomarkers for monitoring MRD.

## Supporting information

S1 Fig The event free survival curves of the whole cohort. The event free survival curves of the whole cohort (dotted line) and patients without treatment related mortality (red line).
(TIF)

S1 File Pediatric T-ALL with *CRLF2* gene expression. (XLSX)

S1 Table Modified St. Jude TXV protocol. (DOCX)

S2 Table The clinical and *CRLF2* expression information of cohort. (DOCX)
