# Supplementary figures and images for: Clinical characterization and prognosis of T cell acute lymphoblastic leukemia with high CRLF2 gene expression in children
Source: PLoS One. 2019 Dec 12;14(12):e0224652. doi: 10.1371/journal.pone.0224652 (PMC6907766; doi:10.1371/journal.pone.0224652)

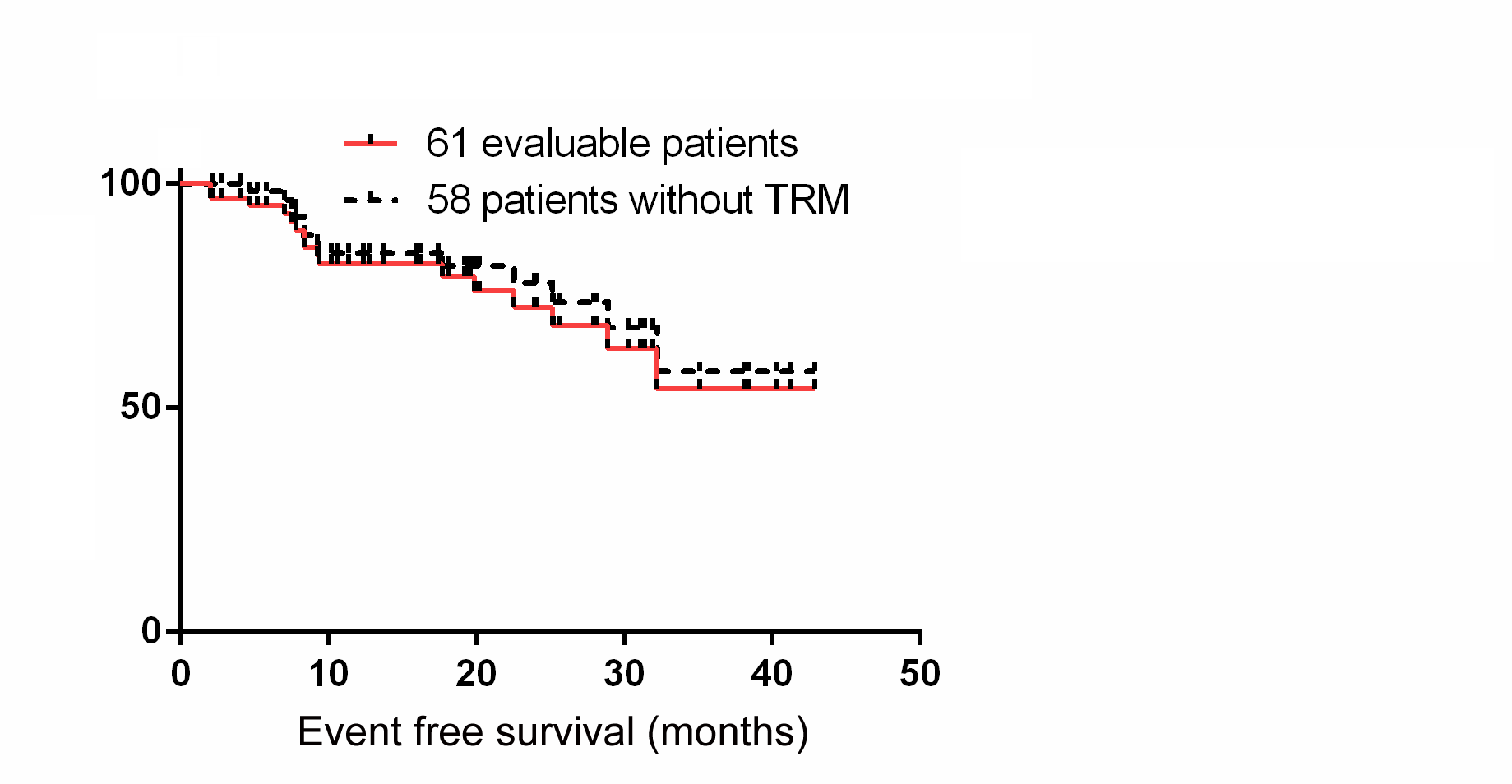

Supplement: S1 Fig — The event free survival curves of the whole cohort (dotted line) and patients without treatment related mortality (red line). (TIF) [file pone.0224652.s001.tif]
