# Supplementary material for: Clinical characterization and prognosis of T cell acute lymphoblastic leukemia with high CRLF2 gene expression in children
Source: PLoS One. 2019 Dec 12;14(12):e0224652. doi: 10.1371/journal.pone.0224652 (PMC6907766; doi:10.1371/journal.pone.0224652)
Supplement: S1 Table — (DOCX) [file pone.0224652.s003.docx]

**S1 Table Modified St. Jude TXV protocol**

| Phase | Drug | Dosage | Duration |
| --- | --- | --- | --- |
| Remission induction | Dex | 8 mg/m^2^/d, po/iv | Days 1-4 |
|  | Pred | 60 mg/m^2^/d, po | Days 5-28, tapering after 9 days |
|  | DNR | 25 mg/m^2^/d, iv | Days 5 and 12 |
|  | VCR | 1.5 mg/m^2^/d (max: 2 mg) iv | Days 5, 12, 19, and 22 |
|  | PEG-asp | 2000 U/m^2^/d, im | Days 6 and 26 |
|  | CTX | 1 g//m^2^/d, iv | Days 29 |
|  | AraC | 50 mg/m^2^, q12h, IH | Days 29 to 35 |
|  | 6-MP | 50 mg/m^2^/d, po | Days 29 to 35 |
|  | TiT | Related to age | Days 5, 8,12, 15, and 19 |
| Consolidation | MTX | 5 g/m^2^/d, iv | Days 1, 15, 29, and 43 |
|  | CF | 15 mg/m^2^, im, q6h, for rescue | 42 h after MTX administration, at least 3 doses |
|  | 6-MP | 25 mg/m^2^/d, po | Days 1 to 56 |
|  | TiT | Related to age | Days 5, 8,12, 15, and 19 |
| Interphase x 5 cycles: every 3 weeks | Dex | 12 mg/m^2^/d, po | Days 1-5 |
|  | DNR | 25 mg/m^2^/d, iv | Day 1 |
|  | VCR | 1.5 mg/m^2^/d (max: 2 mg) iv | Day 1 |
|  | PEG-asp | 2000 U/m^2^/d, im | Day 3 |
|  | 6-MP | 25 mg/m^2^/d, po | Days 1 to 21 |
|  | TiT | Related to age | Day 1 |
| Reinduction of remission | Dex | 8 mg/m^2^/d, po | Days 1 to 7 and 15 to 21 |
|  | VCR |  | Days 1, 8, and 15 |
|  | PEG-asp | 2000 U/m^2^/d, im | Day 3 |
|  | AraC | 2 g/m^2^, q12h | Days 1 and 2 |
|  | TiT | Related to age | Day 1 |
| Maintenance |  |  |  |
| Course-1 (5 cycles): every 4 weeks | MTX | 25 mg/m^2^/d, po | Day 1 and 8 |
|  | 6-MP | 50 mg/m^2^/d, po | Days 1 to 14 |
|  | CTX | 300 mg/m^2^/d, iv | Day 15 |
|  | VCR | 1.5 mg/m^2^/d (max: 2 mg) iv | Day 15 |
|  | AraC | 300 mg/m^2^/d, iv | Day 15 |
|  | DEX | 8 mg/m^2^/d, po | Days 15 to 21 |
|  | TiT | Related to age | Day 15 |
| Course-2 (7 cycles): every 8 weeks | MTX | 25 mg/m^2^/d, po | Days 1, 8, 15, 22, 29, 36, and 42 |
|  | 6-MP | 50 mg/m^2^/d, po | Days 1 to 49 |
|  | CTX | 300 mg/m^2^/d, iv | Day 50 |
|  | VCR | 1.5 mg/m^2^/d (max: 2 mg) iv | Day 50 |
|  | AraC | 300 mg/m^2^/d, iv | Day 50 |
|  | DEX | 8 mg/m^2^/d, po | Days 50 to 56 |
|  | TiT | Related to age | Day 50 |
| Course-3 (2 cycles) | MTX | 25 mg/m^2^/d, po | Days 1 to 56 |
|  | 6-MP | 50 mg/m^2^/d, po | Days 1 to 56 |
