# Supplementary material for: Clinical characterization and prognosis of T cell acute lymphoblastic leukemia with high CRLF2 gene expression in children
Source: PLoS One. 2019 Dec 12;14(12):e0224652. doi: 10.1371/journal.pone.0224652 (PMC6907766; doi:10.1371/journal.pone.0224652)
Supplement: S2 Table — (DOCX) [file pone.0224652.s004.docx]

**S2 Table The clinical and CRLF2 expression information of cohort**

| **Pt.** | **Gender** | **Age at diagnosis (m)** | **WBC**  **(×10^9^/L)** | **Karyotype** | **Fusion Genes** | **CRLF2 level** | **CRLF2 expression (%)** | **Mediastinal mass^a^** | **TP1 BM smear** | **TP1 MRD^d^** | **TP2 BM smear** | **TP2 MRD^d^** |
| --- | --- | --- | --- | --- | --- | --- | --- | --- | --- | --- | --- | --- |
| 1^b^ | female | 60 | 61.97 | Normal | *BCR/ABL1* | high | 96.87 | Y | NA | NA | NA | NA |
| 2 | male | 62 | 330.52 | Abnormal | NA | low | 75.30 | Y | M2 | 0.3683 | CR | 0.0581 |
| 3 | male | 109 | 384.05 | Abnormal | *SIL/TAL1* | high | 96.39 | N | M1 | 0.2181 | CR | 0.0258 |
| 4 | male | 178 | 23.56 | Normal | *MLL/ENL* | low | 75.30 | Y | M1 | 0.3879 | CR | 0.0235 |
| 5 | female | 148 | 1.21 | Normal | NA | high | 93.39 | N | M3 | 0.9493 | CR | 0.0225 |
| 6 | female | 166 | 3.65 | Normal | NA | low | 67.55 | N | M1 | 0.6552 | CR | 0.0202 |
| 7 | male | 123 | 3.2 | Abnormal | NA | high | 99.4 | N | M2 | 0.3384 | CR | 0.0102 |
| 8 | male | 175 | 34.53 | Normal | NA | high | 92.78 | N | M2 | 0.491 | CR | 0.0054 |
| 9 | male | 113 | 57.83 | Normal | NA | low | 87.22 | Y | M1 | 0.3785 | CR | 0.0045 |
| 10 | male | 97 | 247.47 | Abnormal | *SIL/TAL1* | low | 17.09 | N | M1 | 0.416 | CR | 0.0033 |
| 11 | male | 141 | 1.32 | No detection | NA | high | 95.64 | N | M3 | 0.748 | CR | 0.0011 |
| 12 | female | 39 | 191.17 | Normal | NA | high | 90.76 | Y | M2 | 0.32 | CR | 0 |
| 13 | male | 86 | 15.07 | Normal | NA | low | 11.64 | N | M2 | 0.2289 | CR | 0 |
| 14 | male | 67 | 62.51 | Normal | NA | low | 32.22 | N | M1 | 0.195 | CR | 0 |
| 15 | female | 94 | 1.93 | Normal | NA | high | 91.28 | N | M1 | 0.164 | CR | 0 |
| 16 | male | 157 | 1.08 | No detection | NA | high | 92.17 | N | M1 | 0.1443 | CR | 0 |
| 17 | male | 84 | 392.41 | Normal | *SIL/TAL1* | high | 92.77 | Y | M1 | 0.0713 | CR | 0 |
| 18 | male | 53 | 123.06 | Normal | NA | low | 49.40 | Y | M1 | 0.0692 | CR | 0 |
| 19 | male | 64 | 329.32 | Normal | *SIL/TAL1* | low | 47.28 | Y | M1 | 0.042 | CR | 0 |
| 20 | male | 134 | 33.8 | Normal | NA | high | 95.33 | N | M1 | 0.0264 | CR | 0 |
| 21 | female | 166 | 5.97 | Abnormal | NA | low | 82.11 | N | M1 | 0.0223 | CR | 0 |
| 22 | male | 125 | 434.72 | Normal | *SIL/TAL1* | low | 72.14 | Y | M1 | 0.0196 | CR | 0 |
| 23 | male | 57 | 248.95 | Normal | *SIL/TAL1* | low | 22.57 | Y | M1 | 0.0146 | CR | 0 |
| 24 | male | 91 | 9.69 | Normal | *SIL/TAL1* | low | 15.62 | Y | M1 | 0.0136 | CR | 0 |
| 25 | male | 102 | 154.03 | Normal | *SIL/TAL1* | low | 51.81 | N | M1 | 0.0117 | CR | 0 |
| 26 | male | 118 | 404.33 | Normal | *SIL/TAL1* | low | 69.28 | Y | M1 | 0.0057 | CR | 0 |
| 27 | male | 90 | 66.92 | Normal | NA | low | 9.40 | Y | M1 | 0.0036 | CR | 0 |
| 28 | female | 62 | 12.81 | Abnormal | NA | low | 42.64 | Y | M1 | 0.003 | CR | 0 |
| 29 | male | 99 | 4.34 | Normal | NA | low | 60.24 | Y | M1 | 0.0021 | CR | 0 |
| 30 | female | 154 | 74.29 | Abnormal | NA | high | 97.56 | Y | M1 | 0.0016 | CR | 0 |
| 31 | male | 121 | 5.16 | Normal | NA | low | 36.89 | N | M1 | 0.0012 | CR | 0 |
| 32 | male | 61 | 72.13 | Normal | *SIL/TAL1* | high | 92.17 | N | M1 | 0 | CR | 0 |
| 33 | male | 44 | 232.03 | Normal | *SIL/TAL1* | high | 92.58 | N | M1 | 0 | CR | 0 |
| 34 | male | 171 | 46.43 | Normal | *SIL/TAL1* | high | 95.18 | Y | M1 | 0 | CR | 0 |
| 35 | male | 127 | 4.29 | Normal | NA | high | 88.42 | Y | M1 | 0 | CR | 0 |
| 36 | male | 151 | 325.21 | Normal | NA | high | 95.18 | Y | M1 | 0 | CR | 0 |
| 37 | female | 19 | 543.79 | Normal | NA | high | 91.57 | Y | M1 | 0 | CR | 0 |
| 38 | female | 20 | 508.71 | Normal | *SIL/TAL1* | high | 92.77 | Y | M1 | 0 | CR | 0 |
| 39 | male | 117 | 228.87 | Normal | NA | low | 12.02 | N | M1 | 0 | CR | 0 |
| 40 | male | 127 | 320.41 | Abnormal | NA | low | 35.54 | N | M1 | 0 | CR | 0 |
| 41 | female | 41 | 90.24 | Abnormal | NA | low | 72.89 | N | M1 | 0 | CR | 0 |
| 42 | male | 147 | 508.15 | Normal | NA | low | 71.69 | N | M1 | 0 | CR | 0 |
| 43 | male | 25 | 199.27 | Normal | NA | low | 77.71 | N | M1 | 0 | CR | 0 |
| 44 | male | 176 | 106.67 | Normal | NA | low | 43.98 | N | M1 | 0 | CR | 0 |
| 45 | female | 139 | 406.7 | Normal | *SIL/TAL1* | low | 84.94 | N | M1 | 0 | CR | 0 |
| 46 | female | 125 | 3.13 | Normal | NA | low | 26.60 | N | M1 | 0 | CR | 0 |
| 47 | male | 49 | 74.58 | Normal | NA | low | 51.20 | Y | M1 | 0 | CR | 0 |
| 48 | male | 123 | 133.29 | Abnormal | NA | low | 60.24 | Y | M1 | 0 | CR | 0 |
| 49 | male | 106 | 22.02 | Abnormal | NA | low | 59.64 | Y | M1 | 0 | CR | 0 |
| 50 | male | 69 | 50.31 | Abnormal | NA | low | 10.84 | Y | M1 | 0 | CR | 0 |
| 51 | male | 84 | 14.72 | Normal | *SET/CAN* | low | 53.69 | Y | M1 | 0 | CR | 0 |
| 52 | male | 73 | 41.98 | Normal | NA | low | 2.96 | Y | M1 | 0 | CR | 0 |
| 53 | male | 24 | 95.7 | Normal | NA | low | 56.03 | Y | M1 | 0 | CR | 0 |
| 54 | male | 81 | 9.83 | Normal | *SIL/TAL1* | low | 71.69 | Y | M1 | 0 | CR | 0 |
| 55 | male | 150 | 75.3 | Normal | *SIL/TAL1* | low | 21.69 | Y | M1 | 0 | CR | 0 |
| 56 | male | 69 | 23.78 | Normal | NA | low | 46.37 | Y | M1 | 0 | CR | 0 |
| 57 | male | 47 | 47.52 | Normal | NA | low | 53.01 | Y | M1 | 0 | CR | 0 |
| 58 | male | 125 | 61.85 | Normal | NA | low | 50.60 | Y | M1 | 0 | CR | 0 |
| 59 | male | 93 | 10.52 | Normal | NA | low | 87.35 | Y | M1 | 0 | CR | 0 |
| 60 | male | 44 | 4.21 | No detection | NA | low | 34.67 | N | M1 | 0.0024 | CR | 0 |
| 61 | male | 66 | 62.04 | Normal | NA | high | 91.25 | Y | M2 | 0.076 | CR | 0 |
| 62 | male | 53 | 68.12 | Normal | NA | high | 90.60 | Y | M3 | 0.118 | CR | 0 |
| 63^c^ | male | 44 | 166.06 | Normal | NA | low | 46.91 | Y | M1 | 0 | NA | NA |

Pt. patient; NA, with no positive fusion gene signal among the fusion genes been detected;

a. Y yes, N no; b. this patient died at the 1st week of diagnosis, no further detection was performed; c. this patient withdrawn from the treatment for family reason and no further detection or contact were performed; d. the value of MRD ≥ 0.0001 was considered positive
